# Loss-adjusted survival of cervix cancer in Khon Kaen, Northeast Thailand

**DOI:** 10.1038/sj.bjc.6601959

**Published:** 2004-06-15

**Authors:** S Sriamporn, R Swaminathan, D M Parkin, S Kamsa-ard, M Hakama

**Affiliations:** 1 Department of Epidemiology, Faculty of Public Health, Khon Kaen University, Khon Kaen 40002, Thailand; 2 Cancer Institute (WIA), Chennai, India; 3 International Agency for Research on Cancer, Lyon, France; 4 Cancer Unit, Srinagarind Hospital, Khon Kaen University, Thailand; 5 Tampere School of Public Health, University of Tampere, Finland

**Keywords:** cervix cancer, survival, loss to follow-up

## Abstract

For incident cancers of the cervix uteri (601 cases) registered in the population-based cancer registry of Khon Kaen province, Northeast Thailand, in 1985–1990 loss-adjusted survival probabilities were estimated by a logistic regression model with four prognostic factors (age at diagnosis, stage of disease, place of residence and treatment), and compared with observed survival, estimated by the actuarial method. All patients were followed up for a minimum of 5 years, using both passive and active methods. In all, 27.6% of patients were lost to follow-up within 5 years of the index date. The overall observed survival at 5 years was 56.8% and loss-adjusted survival was 54.7%. The difference between the loss-adjusted and observed survival at 5 years was small: 2.1% overall, varying between 0.8 and 3.5 percent units for any prognostic group. The assumption of independence of loss to follow-up and death in the calculation of survival by the actuarial method in this, and probably in other, population-based series, is reasonable and leads to no material bias in the estimates.

Population level survival is usually estimated by the life-table method, in which cumulative probability of survival is calculated at successive annual intervals after diagnosis (Cutler & Ederer, 1958; Ederer *et al*, 1961). Information from all cases is used, including cases whose follow-up ends due to closure of the study, and those lost to follow-up before closure. Survival estimates may be biased if the proportion of cases lost to follow-up is substantial (as in many developing countries, where health information systems are not well developed), and if the loss to follow-up is correlated with the probability of death (prognosis) of the patient after he or she was lost.

Prognostic factors that may also predict loss to follow-up are related to the clinical characteristics of the disease, the patient and the social environment. For example, recurrence or relapse of the disease and serious comorbidity are prognostic factors that may cause the patient to move away (for treatment, or terminal care), making them impossible to trace. Social status influences the probability of survival from cancer (Kogevinas and Porta, 1997) and may also affect the ability to follow-up of a subject.

Information on the association between prognostic factors and loss to follow-up can be used to reduce the bias in estimates of survival (Ganesh, 1995; Mathew, 1996). In this paper, we calculate the absolute survival of cases of cancer of the cervix recorded by a population-based cancer registry in Thailand, using the actuarial method, and examine the effect of adjustment for differential loss to follow-up within subgroups of patients at different risk of death from the disease (‘loss adjustment’).

## SUBJECTS AND METHODS

A total of 630 invasive incident cancers of the cervix were registered during 1985–1990 in the population-based cancer registry covering the province of Khon Kaen. Of these, 29 (4.6%) were registered on the basis of a death certificate only, and were excluded from the survival analysis. For the remaining 601 cases, data on age at diagnosis, area (district) of residence, date of incidence, topography, morphology, stage of disease, treatment (whether treatment by surgery, radiation or chemotherapy was recorded in the patient file), date and vital status (alive or dead) at last contact were abstracted from the registry database.

Patients were followed up until death, or date of loss to follow-up, or 31 December 1995 (closing date). Therefore, the potential length of follow-up was 5–10 years. The registry used both passive and active measures to establish the vital status (alive/dead) of cancer patients.

### Passive follow-up

All death certificates (with a mention of any cancer (ICD-9: 140–208) as underlying or contributing cause of death) were obtained from the Provincial Health Department. The death certificates were linked to the cancer registry database at annual intervals (using national ID number, name, date of birth and address) and the date of death updated for matching cases.

### Active follow-up

For the remaining unmatched cases, information on follow-up was collected by visiting the various hospitals to scrutinise case records, and by making enquiries of treating physicians and general practitioners. Annual follow-up on the anniversary of the date of incidence was attempted for presumed survivors by sending a reply-paid postcard inquiring about the current status of the patient. If no reply was received, a second postcard was sent to the headman of the village requesting the same information. House visits were also performed wherever feasible.

### Analytical methods

#### Actuarial survival

The estimation of survival probability for each year was carried out by the actuarial method. The index date of this study was the date of incidence. The duration of survival for each case was calculated as the time elapsed from the index date to the date of death or the last date of follow-up or closing date, whichever was earlier. Cumulative absolute survival (Cutler and Ederer, 1958) was estimated using the SURV3 analysis programme (Dickman *et al*, 2002).

#### Loss-adjusted survival

The method proposed by Ganesh (1995) was used and is described in detail in the statistical appendix.

- *Step 1* – Choice of potential confounding (prognostic) factors (*X*_1_,…,*X*_4_), and strata (*j*) for each factor.Cases were allocated to 64 strata within four factors: (i) age (four levels: <40, 40–49, 50–59, 60+), (ii) stage of disease (four levels: I, II, III and IV, unknown), (iii) cancer-directed treatment (two levels: yes, no) and (iv) place of residence (two levels: Muang and surrounding districts, other). Muang district is in the centre of Khon Kaen province where Khon Kaen city is located.
- *Step 2* – Classification of study subjects into two main categories: those with complete follow-up and those lost to follow-up.At a given survival time (annual), ‘*i*’=1–5 (say), the subjects in each stratum (*n*_*ij*_) were classified into two groups: (1) those ‘completely followed up’, denoted by *n*_*ij*_′, comprising those dead (*d*_*ij*_) during the interval (*i*) or alive (*w*_*ij*_) at the end of the annual interval, and (2) those ‘lost to follow-up’, denoted by *l*_*i*_*)*, who were last known to be alive in the annual interval and status unknown thereafter.
- *Step 3* – Computation of probability of death (*q*_*ij*_′) for all *i* and *j* for factors *X*_1_,…,*X*_4_ among cases with complete follow-up (*n*_*ij*_′=*n*_*ij*_−*l*_*i*_).The probability of death (*q*_*ij*_′) at each annual interval *i*, was estimated by means of a logistic regression model, using cases with complete follow-up only (*n*_*ij*_′), with all factors (*j*) taken into account simultaneously in the model. Stata version 7.0 (2001) software was used to estimate the regression coefficients.
- *Step 4* – Computation of expected deaths (*d*_*ij*_′) among cases lost to follow-up (*l*_*ij*_).The expected deaths (*d*_*ij*_′) among the group of cases lost to follow-up (*l*_*ij*_) were estimated by assigning the same probability (*q*_*ij*_′) of death.
- *Step 5* – Computing the loss-adjusted survival for each interval *i*. The computation of the conditional probability of dying (*q*_*i*_), conditional probability of surviving (*p*_*i*_) and the cumulative probability (*P*_*i*_) of surviving the current and subsequent annual intervals of time are estimated by accumulating the numbers *d*_*ij*_, *d*_*ij*_′, *n*_*ij*_, *n*_*ij*_′ over the confounders, *j*, and proceeding under the modified actuarial framework of generating life table, as described in the statistical appendix.

## RESULTS

A total of 601 (95.4%) out of 630 cases of cancer of the cervix diagnosed during 1985–1990 were included in the survival study; all patients were followed to the end of 1995 or later. In all, 83% were diagnosed microscopically. Table 1

**Table 1:** Number of cases, proportion and risk (odds ratio, OR) of death and loss to follow-up at 5 years from the index date and 95% confidence interval (CI) by factors studied

		**Proportion at 5 years from index date**		**Odds ratio (OR) and 95% CI**	
**Factors studied**	**Number of cases**	**Lost (%)**	**Dead (%)**	**Lost OR^a^**	**Dead^b^ OR^a^**
All cases	601	27.6	36.4		

*(1) Age group*					
<40	122	27.9	24.6	1.0	1.0
40–49	194	24.2	35.1	0.9 (0.5–1.5)	1.5 (0.8–2.7)
50–59	158	29.1	36.7	1.2 (0.7–2.0)	2.0 (1.1–3.7)
60+	127	29.9	49.6	1.2 (0.7–2.1)	3.5 (1.8–6.9)

*(2) Stage of diseases*					
Stage I	93	23.7	20.4	1.0	1.0
Stage II	134	28.4	29.1	1.2 (0.7–2.2)	1.8 (0.9–3.6)
Stage III and IV	222	21.6	53.6	0.8 (0.4–1.5)	5.0 (2.7–9.5)
Stage unknown	152	37.5	27.6	1.4 (0.7–2.6)	1.5 (0.7–3.1)

*(3) Treatment*					
Received treatment	428	22.4	36.2	1.0	1.0
No treatment	173	39.9	37.0	2.0 (1.3–3.1)	2.0 (1.2–3.4)

*(4) Residency*					
Muang and surrounding					
Districts	274	28.1	32.9	1.0	1.0
Other districts	327	26.9	39.4	0.8 (0.6–1.2)	1.5 (1.0–2.3)

a ORs of each factor adjusted for all other factors in the table.

b Estimated among those with complete follow-up only.

shows the distribution of cases by age, stage, treatment received and place of residence. In all, 316 cases (53%) were aged under 50 years, and 127 (21%) were over 60; 93 cases (15.5%) were stage I, 22.3% stage II, 31.5% stage III, 5.5% stage IV and 25.3% were of unknown stage at diagnosis. A total of 45.6% of cases were residents of Muang and surrounding districts; 71.2% of patients received treatment through either surgery or radiation or chemotherapy.

### Risk of loss to follow-up and death

The proportion and risk (odds ratio) of death and loss to follow-up at 5 years from the index date, by prognostic factors, are presented in Table 1. The proportion of patients lost to follow-up during the 5-year period was 27.6%, and of dying was 36.4%. The risk of loss to follow-up varied 1.3-fold by age at diagnosis, 1.6-fold by stage of disease and 1.2-fold by place of residence; the risk of loss to follow-up among cases not treated was two-fold higher than those treated.

The risk of death increased 3.5-fold with increasing age at diagnosis, and five-fold with stage of disease (*P*<0.001), with the highest risk observed in stages III and IV. Those with stage unknown also had a higher risk of death than those in stage I (OR=1.5). Patients with no treatment had a two-fold higher risk of death and patients who lived far away from the centre of the province had a 50% higher risk than those who lived nearby.

### Survival from cervix cancer (actuarial and loss-adjusted)

The observed (actuarial) survival at 5 years was 56.8% (Table 2

**Table 2:** Number of cases, proportion lost to follow-up at varying intervals of time and 5-year cumulative absolute and loss-adjusted survival of factors studied

		**% lost to follow-up among persons at risk of death at varying lengths of time (*i*) from index date**			**% Absolute survival**	
**Factors studied**	**No. of cases**	**<1 year**	**1≤*i*<3 years**	**3≤*i*<5 years**	**Actuarial**	**Loss-adjusted^a^**
All cases	601	13.3	5.1	19.3	56.8	54.7

*(1) Age group*						
<40	122	13.1	4.1	17.9	71.0	68.1
40–49	194	11.3	4.8	16.4	59.7	57.8
50–59	158	13.9	6.6	19.8	55.7	53.4
60+	127	15.8	4.8	26.9	38.8	37.6

*(2) Stage of diseases*						
Stage I	93	7.5	1.2	19.4	77.1	74.6
Stage II	134	9.7	9.0	18.3	65.5	63.0
Stage III and IV	222	9.0	5.8	20.2	39.0	38.2
Stage unknown	152	26.3	3.2	19.2	63.3	59.8

*(3) Treatment*						
Received treatment	428	6.8	6.4	17.2	59.2	57.5
No treatment	173	29.5	0.0	27.7	49.9	47.5

*(4) Residency*						
Muang and surrounding districts	274	13.1	5.2	19.6	61.1	58.6
Other districts	327	13.5	5.1	19.0	53.2	51.4

a Each factor adjusted for differential loss to follow-up by other factors in the table.

). During this period, 27.6% of cases were lost to follow-up; 13.3% in the first year, 5.1% of those remaining in the second and third years, and 19.3% of the remainder in the fourth and fifth years (Table 2).

Adjustment for loss of follow-up gave an estimated survival of 54.7% at 5 years from index date, 2.1% units less than the observed (actuarial) survival. This suggests that the patients who were lost to follow-up had a higher mortality than assumed in the actuarial method of survival analysis, in which such deaths occur at the same rate as among those with complete follow-up. Table 2 also gives the estimate of loss-adjusted survival by age group, stage, treatment and residence, each adjusted for differential loss to follow-up by the other three factors.

#### Age

An inverse relationship between survival and age at diagnosis was evident: Patients aged less than 40 years had the best survival and patients aged more than 60 years had the poorest survival by both estimation methods. The degree of bias introduced into the actuarial estimate by differential loss to follow-up was small, in the range of 1.2–2.9% units, and the variation by age was somewhat less than indicated by the actuarial estimates.

#### Stage of disease

Patients with stage I had the best survival (74.6%) and stage III and IV had the poorest survival (38.2%). The reduction in the differences of survival between loss-adjusted and actuarial estimates was the highest in patients with unknown stage (3.5% units) and the smallest in patients with stage III and IV disease (0.8% units).

#### Treatment

Patients who received treatment had better survival (57.5%) than those who had not (47.5%). The reduction in the differences of survival between loss-adjusted survival and actuarial estimates was higher in the untreated (2.4% units) than in those who received treatment (1.7% units).

#### Place of residence

Patients who lived in Muang and surrounding districts had better survival (58.6%) than patients who lived in other districts (51.4%). Loss-adjusted survival revealed a reduction in estimated survival compared with that estimated by the actuarial method, for both residence groups. The difference in survival estimates was 2.5% units for residents of Muang and surrounding districts and 1.8% units for those living in other districts.

These small changes in the estimate of survival following the loss-adjustment procedure indicate the presence of a small bias in the actuarial estimate, resulting from the higher mortality among cases lost to follow-up, than under the actuarial assumption.

## DISCUSSION

The fundamental step in carrying out an end result study is to ensure good and complete follow-up of patients. The actuarial (life table) method uses information from all subjects, including those censored before 5 years follow-up or death. Losses to follow-up and withdrawals may have different effects on the estimates of survival. The actuarial survival rate gives an unbiased estimate of true survival only if censorship has the same distribution between the groups being compared (Hakulinen, 1982) and is independent of risk of the outcome studied (Ganesh, 1995). The bias in the estimation of survival probability is dependent on both the magnitude and nature of losses to follow-up, and may be in either direction. For example, the true probability of death of patients lost to follow-up may be greater than assumed if patients with poor prognosis are more likely to be lost. In these circumstances, the actuarial survival estimate is biased and too high.

If the vital status of all the cases included in a survival study is known at the closing date, the estimation of survival probability by the actuarial method is straightforward and unbiased. In the present study, all subjects could be potentially followed for at least 5 years, so that there were no withdrawals, and all censoring was due to loss to follow-up. In developing countries, it is difficult to obtain complete follow-up information for all patients for various reasons. Typically, cancer patients no longer being followed up in hospital must be traced by active methods, involving postal enquires or home visits. Patients frequently migrate from their usual place of residence to that of their relatives and the hospital/medical centre may not be informed of the change in address. This makes tracing of patients at home difficult, since the new contact address must be obtained from other sources, neighbours or friends, for example. Migration is typically related to the recurrence of the disease, that is, with factors of prognostic significance; its magnitude depends on the nonrandom nature and the extent of the loss to follow-up. It is therefore important in any survival study to ascertain not only the extent of loss to follow-up, but also its independence of the probability of death.

The first step in deciding whether bias in the actuarial estimate of survival is likely is to examine whether loss to follow-up varies according to prognostic variables such as age, stage, residence and treatment group. Computation of loss-adjusted survival (Ganesh, 1995) then takes into consideration such differential losses, by assuming that patients lost to follow-up within strata defined by these variables have the same probability of death as those still remaining under observation and belonging to the same stratum. It is reasonable to expect survival experience in patients lost to follow-up and with complete follow-up to be more similar within a prognostic group, than when all patients are considered together. The difference between the crude actuarial survival and the loss-adjusted value indicates the magnitude of the effect of differential loss to follow-up.

The small difference between the absolute (actuarial) survival and the loss-adjusted survival observed in this study is much less than in other studies (Ganesh, 1995; Mathew, 1996). Large differences in LAR and actuarial estimates have been found in hospital-based series of patients, coming from a wide geographic area, where follow-up of patients no longer attending hospital clinics by house visits was impractical and no postal enquiries were made. In contrast, an international comparison of actuarial and loss-adjusted survival of cervix cancer cases from different population-based cancer registries in developing countries (Swaminathan *et al.* 2002) found that the maximum difference was 4.1%, with a loss to follow-up of 44% and presence of nonrandomness. The observation was not confined to cancer of the cervix; differences for other sites like female breast (data from six registries from developing countries) and larynx (data from Chennai and Mumbai cancer registries) were of similar (small) size. This may be mainly because of the integration of mortality data collection into the case-finding operations of population-based cancer registries (on an annual basis in Khon Kaen). It confirms the finding of the present study, that in a population-based series the assumption of independency of loss to follow-up and death was reasonable, so that calculation of survival by the actuarial method without adjusting for losses to follow-up is likely to have resulted in no material bias in the estimates. However, this is not true in general; the experience of hospital-based series in particular indicates that bias may be considerable, and requires appropriate adjustment of survival estimates.

## STATISTICAL APPENDIX

### LOSS-ADJUSTED SURVIVAL

The procedure for estimating loss-adjusted survival by the stratified method can be described step by step in the actuarial survival estimation framework. However, there is also a different approach which integrates estimation by a regression technique and the life table approach. It is the latter that is sequentially described here:

In the follow-up interval *i* in prognostic stratum *j*, there will be *n*_*ij*_ patients alive at the beginning of the interval, of whom *d*_*ij*_ will die, *w*_*ij*_ will be withdrawn alive because of the closing date of follow-up and *l*_*ij*_ will be lost to follow-up during the interval. Assuming for simplicity that the potential follow-up exceeds *i* intervals, *w*_*ij*_=0. The number with complete follow-up, *n*_*ij*_′, is given by

To overcome the problems in estimation by multifactorial methods caused by sparse numbers in the stratified approach, loss-adjusted survival can also be estimated by logistic regression methods (Breslow and Day, 1980).

The proportion dying with complete follow-up, *q*_*i*_′, given the prognostic factors *x*_*i*_, …, *x*_*k*_, is first estimated for patients not lost to follow-up, *n*_*ij*_′, in the interval *i*:

where , the hazard in interval *i* given the prognostic factors. The proportion of deaths can be estimated for each level of any prognostic variable *x*_*i*_ adjusting for the effect of the other prognostic variables. This is done for every interval *i*.

The expected number of deaths among patients lost to follow-up is then computed as *d′*_*ij*_*=q′*_*ij*_*l*_*ij*_, and the expected proportion of deaths among all *n*_*ij*_ cases is

The procedure is repeated for the next interval (*i=i*+1) with  and with  and with  and for the other prognostic strata.

Accumulating over prognostic strata will result in an annual loss-adjusted rate:

and the cumulative loss-adjusted (crude) survival rate is

If there are withdrawals (*w*_*ij*_), the normal actuarial approximation is

The approach corresponds to adjustment by stratification.

The process described for the stratified analysis will be applied for *d*_*ij*_′, *q*_*i*_(LAR) and ultimately for *P*_i_(LAR), by repeating the estimation of *q*_*i*_(LAR) over the first *i* intervals.
